# Automated tumor regression grade assessment and survival prediction in esophageal cancer via weakly supervised multiple instance learning

**DOI:** 10.3389/fmed.2026.1751768

**Published:** 2026-02-02

**Authors:** Zhengjin Liu, Lin Zhao, Ziqing Zhao, Wenjuan Qin, Jun Zhong, Fenglian Lin, Meizhen Lu, Ruixiang Guo, Qunhuang Guo, Hui Xu, Shouguo Li, Hao Zheng, Haijie Lu

**Affiliations:** 1 Department of Pathology, Zhongshan Hospital of Xiamen University, School of Medicine, Xiamen University, Xiamen, China; 2 Department of Computer Science at School of Informatics, Xiamen University, Xiamen, China; 3 Department of Radiation Oncology, Zhongshan Hospital of Xiamen University, School of Medicine, Xiamen University, Xiamen, China; 4 Department of Radiology, Zhongshan Hospital of Xiamen University, School of Medicine, Xiamen University, Xiamen, China; 5 Department of Health Medicine, Zhongshan Hospital of Xiamen University, School of Medicine, Xiamen University, Xiamen, China; 6 Department of Thoracic Surgery, The First Affiliated Hospital of Anhui Medical University, Hefei, China

**Keywords:** artificial intelligence, multiple instance learning, neoadjuvant therapy, pathology foundation model, tumor regression grade

## Abstract

**Introduction:**

Esophageal cancer remains a major global health burden and is among the leading causes of cancer-related deaths. Accurate evaluation of tumor regression grade (TRG) after neoadjuvant therapy is essential for assessing treatment response and guiding postoperative management. However, conventional TRG assessment relies heavily on subjective histopathological assessments, leading to considerable inter-observer variability and limited reproducibility. We aimed to develop an objective and automated TRG assessment framework using artificial intelligence for digital pathology.

**Methods:**

A retrospective analysis was conducted on 157 patients with esophageal cancer and 1,298 hematoxylin and eosin-stained whole-slide images. Three slide-level pathology foundation models and three multiple instance learning methods were evaluated within a patient-level multiple instance learning framework, enabling weakly supervised TRG prediction based solely on patient-level labels.

**Results:**

The proposed framework achieved a classification accuracy of 82.7% and demonstrated strong agreement with manual pathologist grading. Notably, artificial intelligence-derived TRG score provided superior prognostic stratification compared with conventional assessments, showing significant associations with progression-free and overall survival.

**Discussion:**

This study presents a foundation-model-driven, patient-level multiple instance learning framework for automated evaluation of TRG in esophageal cancer. This approach offers a standardized, reproducible, and clinically interpretable solution that reduces the workload of pathologists and improves prognostic precision. These findings highlight the potential of weakly supervised AI pathology in advancing personalized treatment assessment and decision-making in digital oncology.

## 1 Introduction

Esophageal cancer (EC) is a prevalent malignant tumor of the digestive tract, ranking seventh in incidence and sixth in mortality globally (1). In clinical practice, most patients with EC are diagnosed at locally advanced stages. Because of its poor prognosis, multimodal treatment strategies combining neoadjuvant therapy (NAT) with surgical resection have recently become the standard approach, replacing surgical treatment alone (2–5). The primary objective of NAT is to improve the complete resection rate of tumors and eliminate potential micrometastases through preoperative downstaging, significantly improving patient prognosis (6, 7). Accurate assessment of pathological response after NAT is essential for evaluating therapeutic efficacy and determining optimal postoperative management strategies. Tumor regression grade (TRG) is a crucial histopathological indicator of NAT efficacy and for informing clinical decision-making (8, 9). However, traditional TRG assessments are subjective and rely on visual estimates of the proportion of residual tumor cells relative to fibrosis, or the percentage of residual tumor area within whole-slide images (WSIs) (10). Such manual evaluations are associated with significant inter-observer variability and limited reproducibility. Moreover, pathologists typically need to carefully review dozens, or even hundreds, of histopathological slides to complete the TRG assessment of a single patient with EC, which is extremely time-consuming and imposes a heavy burden on increasingly strained pathologic resources. Therefore, there is an urgent need to develop an objective, accurate, efficient, and reproducible automated tool for evaluating TRG in EC.

The integration of whole-slide imaging with artificial intelligence (AI), particularly deep learning, has recently shown notable potential in various tumor diagnostic and prognostic tasks (11–16). In the field of TRG, several studies have explored the feasibility of AI-assisted assessment following NAT. Tolkach et al. (17) developed an AI system that can be used to automatically detect tumor regions and grade histological regression in esophageal adenocarcinoma, achieving a strong correlation with clinical outcomes and performance comparable to expert pathologists’ assessments. Wang et al. (18) further proposed a semi-supervised knowledge distillation framework to evaluate the pathological response of esophageal squamous cell carcinoma after NAT, demonstrating a strong agreement with pathologists’ assessments of residual tumor percentage. However, their approach required manually labeled image patches and primarily focused on continuous residual tumor quantification rather than direct TRG score prediction.

To address these limitations, we developed an accurate and efficient weakly supervised AI framework for the automated assessment of TRG score after NAT in EC. Compared with the fully supervised methods adopted in previous studies, weakly supervised approaches, such as multiple instance learning (MIL), have emerged as powerful alternatives in computational pathology. As highlighted in a recent comprehensive survey by Waqas et al. (19), MIL has gained significant traction in medical image analysis by drastically reducing the annotation burden while maintaining robust performance across diverse diagnostic tasks. This paradigm enables efficient model training without detailed pixel- or region-level annotations (20–22). Our framework relies solely on patient-level labels provided by pathologists, eliminating the need for precise tumor annotations or manual slide-level grading. Specifically, we first incorporated pathology foundation models into our workflow and systematically compared several state-of-the-art slide-level encoders, including CHIEF (23), Prov-Gigapath (24), and TITAN (25) to identify the optimal feature extractor for WSI representation. Subsequently, we implemented an MIL-based architecture, in which each patient was treated as a “bag” comprising multiple WSIs from the same patient as “instances.” Next, several advanced attention-based MIL algorithms were evaluated to construct a robust patient-level TRG assessment model.

In this study, we demonstrated that AI-predicted TRG score at the patient level were highly consistent with manual pathologist grading across cross-validation folds. Notably, the AI-derived grade exhibited superior prognostic power compared with manual TRG score, indicating the model’s capacity to capture clinically meaningful histological patterns beyond human perception. This framework effectively mitigates the challenges of subjectivity, limited reproducibility, and time-intensive evaluations inherent in traditional TRG assessments. Moreover, by providing enhanced prognostic stratification, it holds strong potential to inform personalized adjuvant treatment decisions for patients with EC, underscoring its clinical applicability and translational significance in the era of pathological AI.

## 2 Materials and methods

### 2.1 Study participants

Eligible patients were aged 18–75 years of either sex, with an Eastern Cooperative Oncology Group performance status of 0–1. All cases were pathologically confirmed as esophageal squamous cell carcinoma, and the patients had radiologically and clinically resectable locally advanced disease that was treated with NAT. Only patients with complete clinicopathological and follow-up data were included in the analyses.

Patients were excluded if they had non-squamous histological subtypes of EC, severe systemic comorbidities that contraindicated standard treatment, or synchronous malignancies in other organs.

### 2.2 Ethical approval

This study was conducted in accordance with the principles of the Declaration of Helsinki. Ethical approval and a waiver of informed consent were obtained from the Ethics Committee of Zhongshan Hospital Affiliated to Xiamen University.

### 2.3 Observation indicators

Histopathological examination was conducted by two experienced pathologists who analyzed the hematoxylin and eosin (H&E)-stained slides to evaluate the resection margins and lymph nodes. Tumor staging was based on the 8th edition of the American Joint Committee on Cancer staging system (26). The tumor response to NAT was assessed using the College of American Pathologists (CAP) grading system (modified Ryan scheme) (8). The specific category-to-criteria mapping is defined as follows: TRG 0 (no viable cancer cells); TRG 1 [single cells or rare small groups of cancer cells, <10% viable residual tumor cells (VRTCs)]; TRG 2 (residual cancer cells outgrown by fibrosis, 10–50% VRTCs); and TRG 3 (minimal or no tumor regression, >50% VRTCs). Patients were categorized as good (TRG 0–1) or poor (TRG 2–3) responders. Overall survival (OS) was defined as the time from diagnosis to death, and progression-free survival (PFS) was defined as the time to tumor progression or death from any cause.

### 2.4 Histopathology data collection and scoring

All hematoxylin and eosin (H&E)-stained sections were digitally scanned and subjected to quality control. The TRG score of the patients was determined based on postoperative pathological assessment criteria and served as the primary source of the labels.

This study included 157 patients with EC who underwent NAT followed by surgical resection. H&E-stained WSIs were retrospectively collected, yielding 1,298 digital pathology slides. Patient-level TRG labels were obtained from pathology reports, and the final TRG score was determined by integrating multiple slides per patient. Given that TRG is inherently a case-level construct and patient-level labels may not represent the histology of individual slides (e.g., a slide with no tumor cells from a TRG 2 patient), direct inheritance of labels is imprecise. Therefore, to enable the evaluation of slide-level feature extraction capabilities, an independent dataset of 120 WSIs was randomly sampled. Two experienced pathologists manually assigned a “slide-level TRG” to each WSI solely based on the proportion of residual tumor area observed on that specific slide, following the CAP criteria. These slide-specific annotations served as the ground truth for the technical benchmarking of foundation models.

### 2.5 Foundation models for slide-level feature extraction

To extract discriminative features from the WSIs, we systematically evaluated three recently developed pathology foundation models: Prov-Gigapath, TITAN, and CHIEF, using 120 WSIs with slide-level TRG annotations.

Prov-Gigapath is a large-scale model trained on 170,000 WSIs. It integrates a tile encoder with a LongNet-based slide encoder to process gigapixel images. While it offers robustness through scale, its architecture is primarily patch-centric. TITAN, in contrast, adopts a multimodal framework (ConCH v1.5 encoder) designed to integrate images and language. Trained on WSIs paired with captions, it excels in cross-modal alignment and zero-shot predictions, focusing on semantic generalization rather than pure histological hierarchy.

CHIEF, however, introduces a hierarchical tokenization mechanism that allows the model to simultaneously capture fine-grained cellular morphology and broader tissue architecture. This architectural characteristic formed our primary *a priori* rationale for selecting CHIEF as the optimal backbone. Accurate TRG grading requires the simultaneous recognition of residual tumor cells (local features) and the surrounding fibrotic stroma (global context). CHIEF’s ability to integrate these multi-scale features makes it theoretically more suitable for TRG assessment compared to the patch-centric approach of Prov-Gigapath or the language-aligned focus of TITAN.

Consequently, CHIEF was selected as the designated feature extractor, with the empirical benchmarking serving primarily to validate this theoretical hypothesis. The pre-trained backbone generates a 768-dimensional representation for each slide. For all experiments, features were extracted using these foundation models at 20× resolution and subsequently subjected to downstream analysis.

For each WSI, features were extracted using all three foundation models at 20× resolution and subsequently underwent further analysis.

### 2.6 Feature visualization

To evaluate whether the learned embeddings reflected TRG-specific differences, we used nonlinear dimensionality reduction methods, namely t-distributed stochastic neighbor embedding and uniform manifold approximation and projection. These techniques project high-dimensional embeddings onto a two-dimensional space, enabling the qualitative visualization of class separation while preserving local neighborhood relationships and global data structures.

### 2.7 Slide-level classification

A quantitative evaluation of slide-level classification was performed using support vector machines trained on embeddings from each foundation model. The support vector machine classifier was chosen for its proven effectiveness in high-dimensional feature spaces and robustness against overfitting. A radial basis function kernel was used to capture the nonlinear class boundaries, and the hyperparameters were optimized within each training fold. To ensure robust estimation of model generalization, five-fold cross-validation was applied, with training and testing sets partitioned at the slide level. The performance metrics included overall accuracy, area under the receiver operating characteristic curve (AUC), precision, recall, *F*_1_-score, and confusion matrices.

### 2.8 Patient-level TRG prediction

Following the comparative evaluation of the foundation models at the slide level, the best-performing foundation model was incorporated into a patient-level predictive framework. Because TRG labels were available only at the case level, we adopted an MIL strategy that treats each patient as a bag and the associated WSIs as instances within that bag. The bag inherits the patient-level TRG label, whereas the labels of the individual instances remain unknown. This approach is particularly suitable for weakly supervised pathology tasks, in which detailed annotations are difficult to obtain.

Three MIL variants are implemented and compared. First, attention-based MIL aggregates slide-level embeddings using an attention mechanism that assigns variable importance weights to each instance, highlighting the most informative slides for prediction. Second, adaptive cross-instance MIL (ACMIL) extends this framework by modeling dependencies among slides and dynamically adjusting the weighting scheme to account for inter-instance relationships. Third, the clustering-constrained attention MIL (CLAM) imposes an additional constraint that partitions instances within a bag into clusters representing positive and negative evidence, thereby enhancing the interpretability and robustness of the aggregated representation. In all three cases, CHIEF was used as the feature extraction backbone because it demonstrated the strongest discriminative power in the preliminary experiments.

Patient-level MIL models were trained and evaluated using five-fold cross-validation, with data split at the patient level to avoid information leakage across the folds. The evaluation metrics included overall accuracy, macro-*F*_1_ score, and AUC, with confusion matrices providing detailed insights into class-specific performance.

### 2.9 Implementation details

For each patient, feature embeddings extracted from all available WSIs (ranging from 2 to 10 slides) were aggregated to form a single patient-level bag, enabling the MIL framework to capture intra-patient histological heterogeneity across different tissue sections. No padding or truncation was applied; all slides belonging to a patient were included during bag aggregation. Five-fold cross-validation was performed with patient-level splits to strictly prevent information leakage between training and test sets.

To address class imbalance between favorable (TRG 0–1) and poor (TRG 2–3) response groups, class-balanced resampling was applied at the patient (bag) level during training. Model optimization was conducted using the AdamW optimizer with an initial learning rate of 2 × 10^−4^ and a weight decay of 4 × 10^−4^. The batch size was set to 2 patients per iteration, and all models were trained for 100 epochs.

A cosine annealing learning rate scheduler with warm-up was employed. The warm-up phase lasted for six epochs with an initial learning rate of 1 × 10^−6^. The cosine schedule was initialized with a cycle length of two epochs, with the cycle length doubling after each restart up to a maximum of 20 epochs. The minimum learning rate was set to 1 × 10^−7^. To ensure stable optimization under weak supervision, each epoch consisted of a fixed number of 200 training iterations.

The training objective was defined as a label-smoothed cross-entropy loss with a smoothing factor of 0.2, which helped mitigate overconfidence and improve generalization in the presence of noisy patient-level supervision. All hyperparameters were kept identical across cross-validation folds, resulting in negligible variation and ensuring reproducible model training.

### 2.10 Statistical analysis

Statistical analyses were performed using MATLAB (MathWorks, Natick, MA, United States). Model performance was assessed using accuracy, precision, recall, *F*_1_-score, and AUC with 95% confidence intervals (CIs). Group-wise comparisons were performed using paired *t*-tests or Wilcoxon signed-rank tests, with significance set at *p* < 0.05. Uniform manifold approximation and projection were performed for feature visualization and implemented using MATLAB.

Deep learning experiments, including feature extraction from foundation models and MIL frameworks, were conducted in Python (version 3.10) using an NVIDIA GTX 3090 GPU.

## 3 Results

### 3.1 Baseline characteristics of study participants

We analyzed 157 patients with esophageal squamous cell carcinoma who received NAT treatment. Among them, 126 (80.3%) were male, and 100 (63.7%) were aged ≥60 years. Postoperative pathological outcomes showed that 41 and 116 patients achieved TRG 0–1 and TRG 2–3, respectively. The baseline characteristics of the patients are summarized in Table 1.

**Table 1:** Baseline characteristics of patients with esophageal squamous cell carcinoma receiving neoadjuvant therapy.

Characteristic	*N*	(%)
Age (years)		
<60	57	36.3%
≥60	100	63.7%
Sex		
Male	126	80.3%
Female	31	19.7%
BMI		
<18	31	19.8%
18–24	105	66.8%
>24	21	13.4%
Tumor location		
Upper	14	9.0%
Middle	93	59.2%
Lower	50	31.8%
Clinical stage		
II	39	24.8%
III	102	65.0%
IVa	16	10.2%
Neoadjuvant therapy cycle		
≤2	121	77.1%
>2	36	22.9%
ypT stage		
T0–T2	73	46.5%
T3–T4	84	53.5%
ypN stage		
N0	91	58.0%
N1–N3	66	42.0%
TRG		
TRG 0–1	41	26.1%
TRG 2–3	116	73.9%

TRG, tumor regression grade; BMI, body mass index.

**Figure 1:**
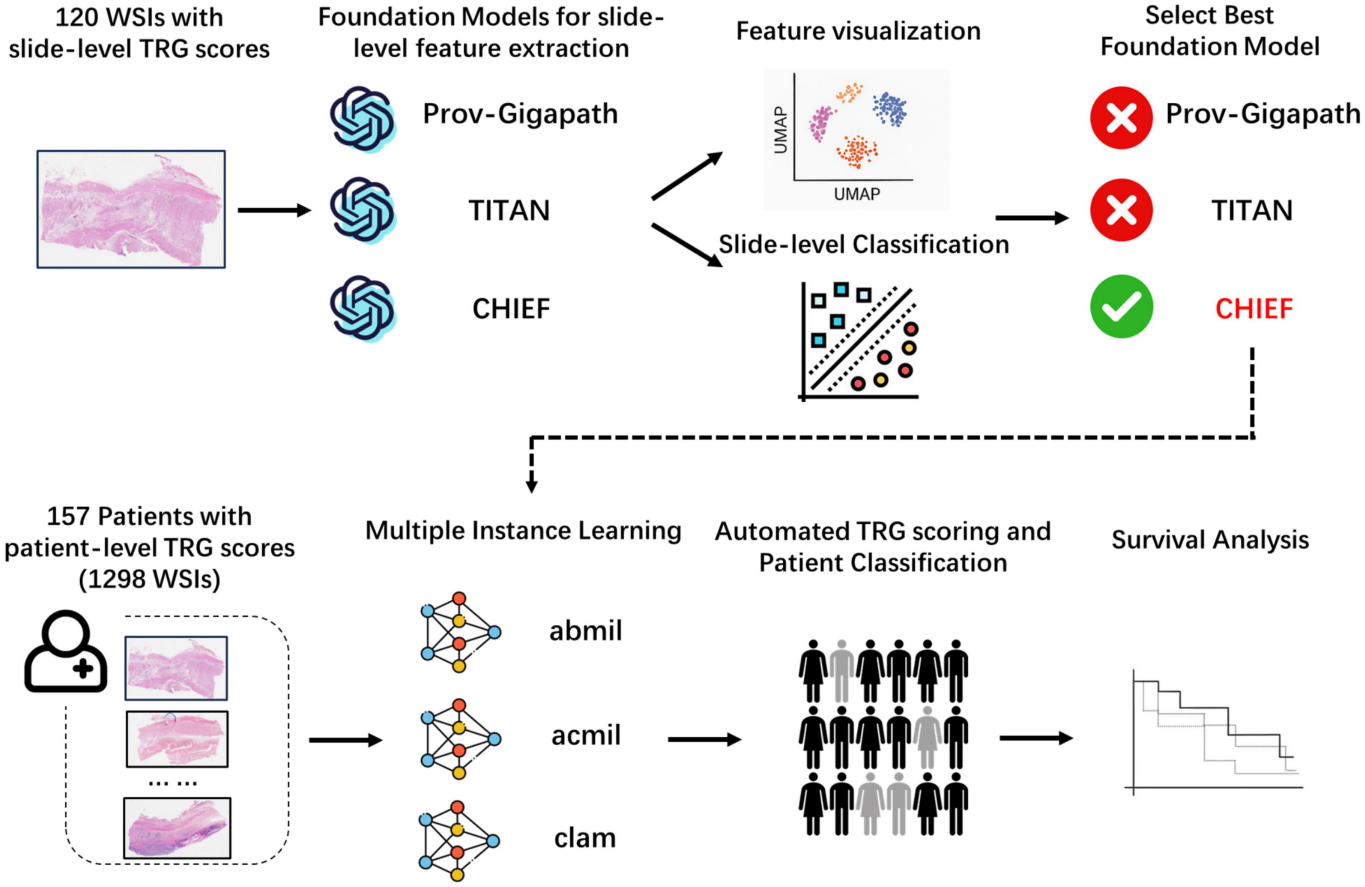
Overall analytical workflow for tumor regression grade prediction in esophageal cancer. Schematic illustration of the two-stage pipeline. In stage 1, slide-level embeddings were extracted from H&E WSIs using three pathology foundation models (CHIEF, Prov-Gigapath, and TITAN) and benchmarked using a five-fold cross-validated support vector machine (SVM) classification. In stage 2, the best-performing model (CHIEF) provided slide-level representations for patient-level MIL frameworks (ABMIL, ACMIL, and CLAM), enabling weakly supervised prediction of TRG. Quantitative evaluation and interpretability analyses were performed at the slide and patient levels. H&E, hematoxylin and eosin; WSIs, whole-slide images; MIL, multiple instance learning.

We designed a two-stage analytical workflow to systematically evaluate the performance of the pathology foundation models and establish an interpretable AI framework for TRG score prediction (Figure 1). At the slide level, three large-scale pathology foundation models were evaluated using 120 annotated WSIs to extract high-dimensional histopathological embeddings. The best-performing model was integrated into a patient-level prediction framework based on MIL, in which slide-level embeddings served as instances, and patient-level TRG score served as weak supervisory labels.

**Figure 2:**
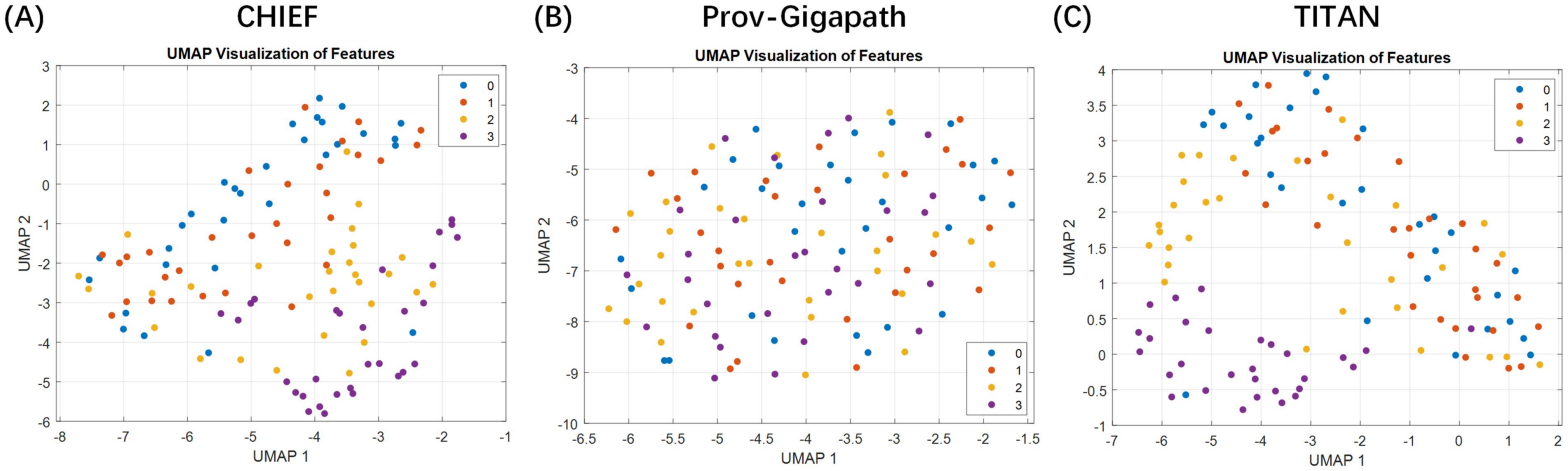
Two-dimensional visualization of slide-level embeddings generated using different pathology foundation models. The plots illustrate the feature space distributions extracted by three distinct backbones: **(A)** CHIEF, **(B)** Prov-Gigapath, and **(C)** TITAN. Method: High-dimensional slide representations (e.g., 768 dimensions for CHIEF) were projected onto a 2D plane using uniform manifold approximation and projection (UMAP). Data representation: Each individual point represents a single whole slide image (WSI) (slide-level), color-coded by its ground-truth TRG category (red: TRG 3; blue: TRG 0; etc.). Takeaway: The visualization demonstrates that the CHIEF model produces the most distinct separation between the extreme classes (TRG 0 vs. TRG 3) with minimal overlap compared to Prov-Gigapath and TITAN, visually validating its selection as the optimal feature extractor for the subsequent patient-level MIL framework.

Furthermore, we projected high-dimensional slide-level embeddings onto 2D using uniform manifold approximation and projection to assess the discriminative capacity of slide-level features extracted with different pathology foundation models (Figure 2). The resulting visualization revealed that the embeddings generated by CHIEF and TITAN demonstrated a clearer separation between the TRG categories, particularly for the extreme classes, such as TRG 0 and TRG 3. In contrast, the features derived from Prov-Gigapath showed substantial overlap across categories, indicating limited discriminability for this specific task. These observations suggest that models pretrained with broader histological diversity (CHIEF) or multimodal objectives (TITAN) were more effective in capturing regression-related morphological variations than the patch-centric Prov-Gigapath model.

The quantitative classification experiments further confirmed these qualitative trends (Table 2). In five-fold cross-validation, CHIEF consistently outperformed the other two models when using support vector machine classifiers trained on slide-level features. It achieved balanced precision, recall, and *F*_1_-scores across most TRG categories, with particularly high performance for TRG 3 (*F*_1_ = 0.933) and acceptable results for intermediate categories such as TRG 1 and TRG 2 (*F*_1_ ranging from 0.556 to 0.746). TITAN achieved competitive performance in TRG 2 and 3 but was less effective in the lower categories. Prov-Gigapath performed poorly overall, with most *F*_1_-scores of 0.40. These findings indicate that CHIEF is the most suitable backbone for downstream patient-level modeling (see Table 3).

**Table 2:** Slide-level classification performance of pathology foundation models.

TRG score	Precision	Recall	*F*_1_-score
CHIEF			
0	0.686	0.801	0.738
1	0.625	0.500	0.556
2	0.733	0.759	0.746
3	0.933	0.933	0.933
Prov-Gigapath			
0	0.286	0.333	0.308
1	0.133	0.067	0.089
2	0.242	0.267	0.254
3	0.361	0.448	0.400
TITAN			
0	0.565	0.433	0.491
1	0.444	0.533	0.485
2	0.645	0.667	0.656
3	0.931	0.931	0.931

**Table 3:** Patient-level TRG prediction performance of multiple instance learning (MIL) models.

	ABMIL			ACMIL			CLAM		
Class	AUC	95% CI lower	95% CI upper	AUC	95% CI lower	95% CI upper	AUC	95% CI lower	95% CI upper
Fold 1									
TRG 0	0.918	0.817	1.000	0.866	0.695	1.000	0.970	0.903	1.000
TRG 1	1.000	1.000	1.000	0.903	0.795	0.994	0.968	0.898	1.000
TRG 2	0.689	0.332	0.971	0.748	0.414	1.000	0.696	0.425	0.964
TRG 3	0.914	0.800	0.988	0.886	0.765	1.000	0.894	0.758	0.980
Macro-average	0.880	0.699	0.964	0.851	0.719	0.976	0.882	0.755	0.987
Micro-average	0.899	0.790	0.963	0.868	0.772	0.973	0.904	0.804	0.967
Fold 2									
TRG 0	0.964	0.839	1.000	0.973	0.875	1.000	0.973	0.903	1.000
TRG 1	0.800	0.578	0.971	0.967	0.894	1.000	0.750	0.405	1.000
TRG 2	0.698	0.397	0.937	0.620	0.410	0.889	0.604	0.398	0.784
TRG 3	0.750	0.599	0.910	0.782	0.619	0.947	0.762	0.587	0.931
Macro-average	0.803	0.680	0.937	0.835	0.695	0.921	0.772	0.626	0.899
Micro-average	0.828	0.732	0.909	0.846	0.736	0.914	0.826	0.707	0.907
Fold 3									
TRG 0	0.962	0.862	1.000	0.969	0.889	1.000	0.969	0.877	1.000
TRG 1	0.714	0.345	1.000	0.690	0.378	0.970	0.869	0.716	1.000
TRG 2	0.613	0.259	0.920	0.727	0.286	1.000	0.667	0.375	0.981
TRG 3	0.857	0.671	0.974	0.882	0.744	0.991	0.887	0.759	0.973
Macro-average	0.787	0.639	0.911	0.817	0.708	0.955	0.848	0.747	0.931
Micro-average	0.862	0.755	0.938	0.895	0.813	0.984	0.888	0.768	0.964
Fold 4									
TRG 0	0.931	0.778	1.000	0.977	0.907	1.000	1.000	1.000	1.000
TRG 1	0.828	0.633	1.000	0.914	0.782	1.000	0.966	0.915	1.000
TRG 2	0.815	0.631	0.965	0.755	0.565	0.985	0.772	0.519	1.000
TRG 3	0.946	0.853	1.000	0.913	0.774	1.000	0.900	0.724	1.000
Macro-average	0.880	0.774	0.977	0.890	0.770	0.981	0.909	0.804	1.000
Micro-average	0.891	0.793	0.964	0.916	0.844	0.977	0.940	0.867	0.992
Fold 5									
TRG 0	0.905	0.813	1.000	0.917	0.773	1.000	0.964	0.869	1.000
TRG 1	0.454	0.138	0.762	0.585	0.267	0.893	0.592	0.267	0.907
TRG 2	0.877	0.759	0.995	0.709	0.500	0.877	0.727	0.535	0.889
TRG 3	0.969	0.889	1.000	0.939	0.846	1.000	0.939	0.833	1.000
Macro-average	0.801	0.707	0.875	0.787	0.681	0.883	0.806	0.657	0.932
Micro-average	0.833	0.720	0.960	0.830	0.732	0.905	0.812	0.691	0.902
Overall									
TRG 0	0.897	0.824	0.957	0.899	0.814	0.964	0.955	0.899	0.992
TRG 1	0.707	0.516	0.847	0.735	0.567	0.868	0.771	0.628	0.879
TRG 2	0.749	0.627	0.856	0.737	0.627	0.839	0.693	0.584	0.776
TRG 3	0.877	0.814	0.939	0.870	0.799	0.919	0.859	0.804	0.913
Macro-average	0.807	0.746	0.865	0.810	0.745	0.868	0.820	0.771	0.880
Micro-average	0.860	0.817	0.913	0.873	0.828	0.911	0.870	0.829	0.904

TRG, tumor regression grade; AUC, area under the receiver operating characteristic curve.

Based on the slide-level results, we constructed a patient-level TRG prediction framework using MIL. The dataset comprised 157 patients, each contributing 2–10 WSIs. Because only patient-level TRG labels were available, each patient was treated as a *bag* containing multiple *instances* (WSIs), with the CHIEF-extracted features of each slide serving as instance representations.

We implemented and compared three representative MIL approaches, namely attention-based MIL (ABMIL), ACMIL, and CLAM, to aggregate slide-level features into patient-level predictions. The model was trained using a five-fold cross-validation setting to ensure patient-level independence between the folds.

Across the five-fold cross-validation, the models demonstrated robust stability. We report the performance as the mean metrics across the folds. The MIL variants achieved a mean macro-average AUC of 0.810 (95% CI: 0.745–0.865) for ACMIL and 0.820 (95% CI: 0.771–0.880) for CLAM. The mean micro-average AUCs consistently exceeded 0.86. Specifically, the CLAM model achieved the highest mean macro-average AUC, while ACMIL demonstrated the most balanced performance with a mean overall accuracy of 82.7%.

**Figure 3:**
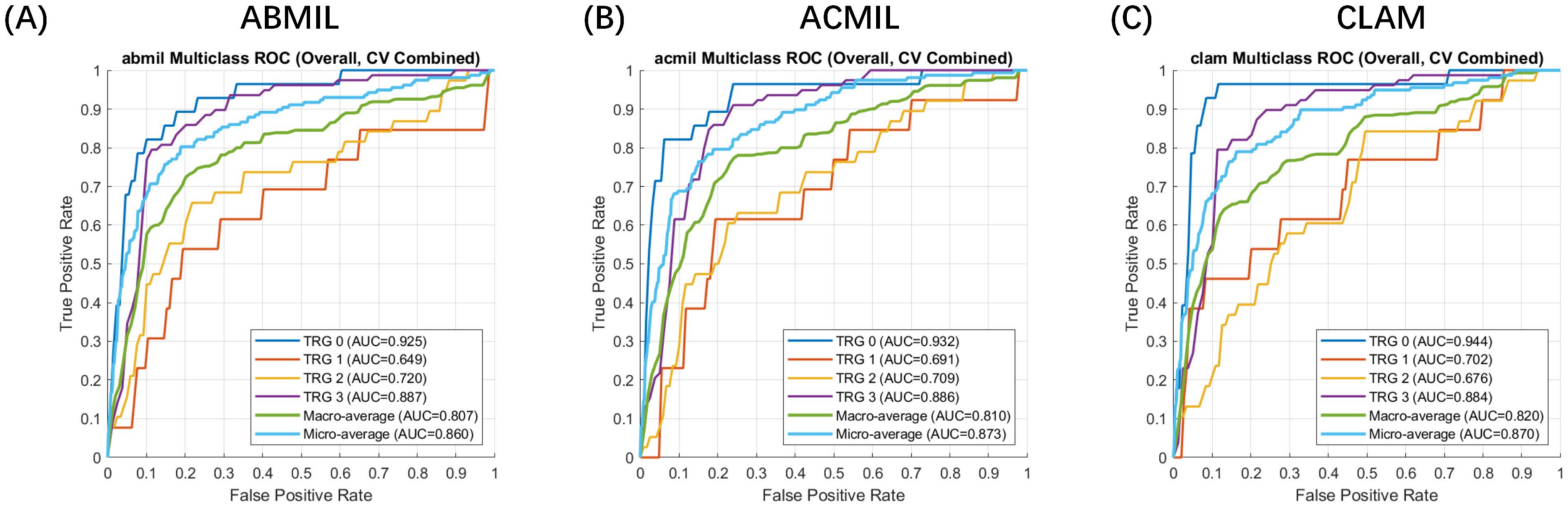
Receiver operating characteristic (ROC) curve analysis of patient-level multiple instance learning models for tumor regression grade prediction. Evaluation setting: The curves represent the patient-level classification performance evaluated using a 5-fold cross-validation scheme. Feature embeddings were extracted using the CHIEF foundation model. Metrics: The plots display the macro-average (blue) and micro-average (orange) ROC curves for the multi-class prediction (TRG 0–3). Model comparison: **(A)** ABMIL, **(B)** ACMIL, and **(C)** CLAM. The shaded areas (if applicable) or curves represent the mean performance across folds, demonstrating the robust discriminative ability of the MIL frameworks, with CLAM and ACMIL showing superior area under the curve (AUC) values.

The overall receiver operating characteristic curves (Figure 3) illustrated clear separability across the TRG categories, with TRG 0 and 3 achieving the highest discriminative power (AUCs of 0.955 and 0.859 for CLAM, respectively). Intermediate grades (TRG 1–2) showed moderate but consistent classification performance (AUCs = 0.693–0.771).

**Figure 4:**
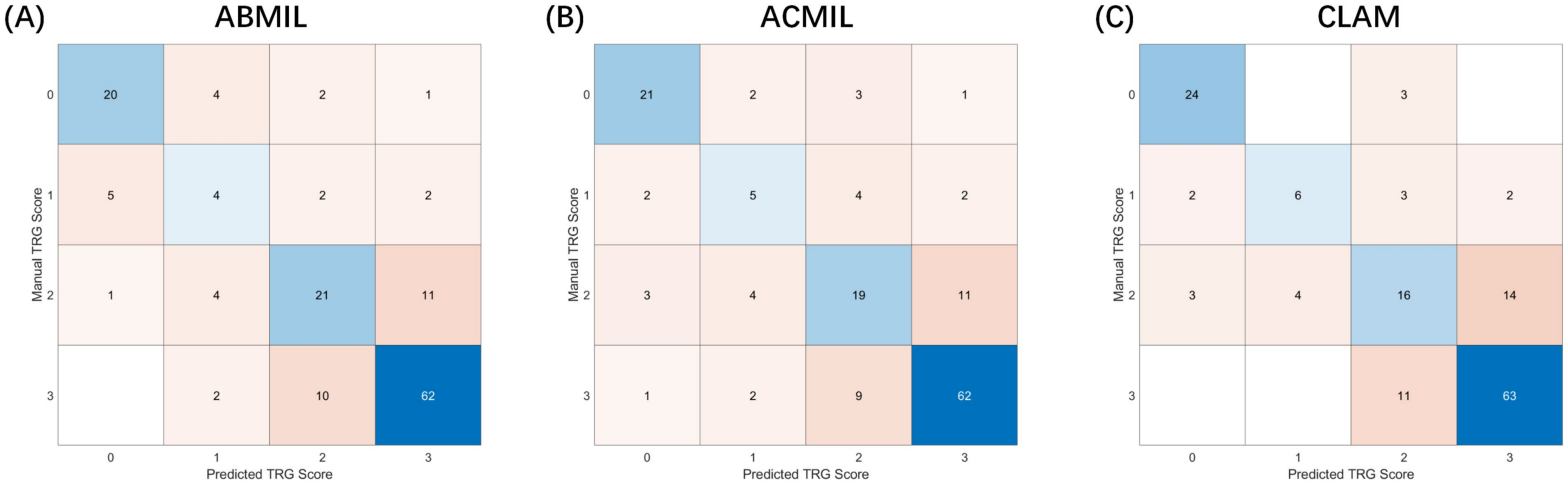
Confusion matrices of patient-level tumor regression grade classification. Experimental setup: The matrices visualize the classification results on the test sets accumulated across 5-fold cross-validation. Input features were extracted using the CHIEF backbone. Axes definition: The *Y*-axis represents the manual TRG score (ground truth) assigned by pathologists, while the *X*-axis represents the predicted TRG score generated by the AI models. Values: Numbers inside the cells indicate the raw count of patients. Darker colors represent higher density/agreement. Comparison: **(A)** ABMIL, **(B)** ACMIL, and **(C)** CLAM. Notably, the ACMIL model **(B)** demonstrates the strongest diagonal alignment (highest agreement with human graders) and the most balanced classification accuracy (82.7%) compared to other variants.

The patient-level confusion matrices (Figure 4) revealed that most classification errors occurred between adjacent TRG categories, reflecting the inherent ambiguity in histological response assessment. Across all three MIL models, predictions of TRG 0 and TRG 3 showed high agreement with the ground truth (accuracy >0.85), whereas TRG 1 and TRG 2 were more prone to misclassification owing to overlapping morphological features such as mixed residual tumor and stromal fibrosis.

Notably, the ACMIL model demonstrated the most balanced performance, correctly classifying 84.6% of the TRG 0/1 cases and 80.3% of the TRG 2/3 cases, yielding an overall accuracy of 82.7%. The confusion matrices further revealed that the MIL models captured the ordinal structure of TRG grading, as most mispredictions deviated by only one grade, underscoring the biological continuity between adjacent TRG levels rather than the random errors.

**Figure 5:**
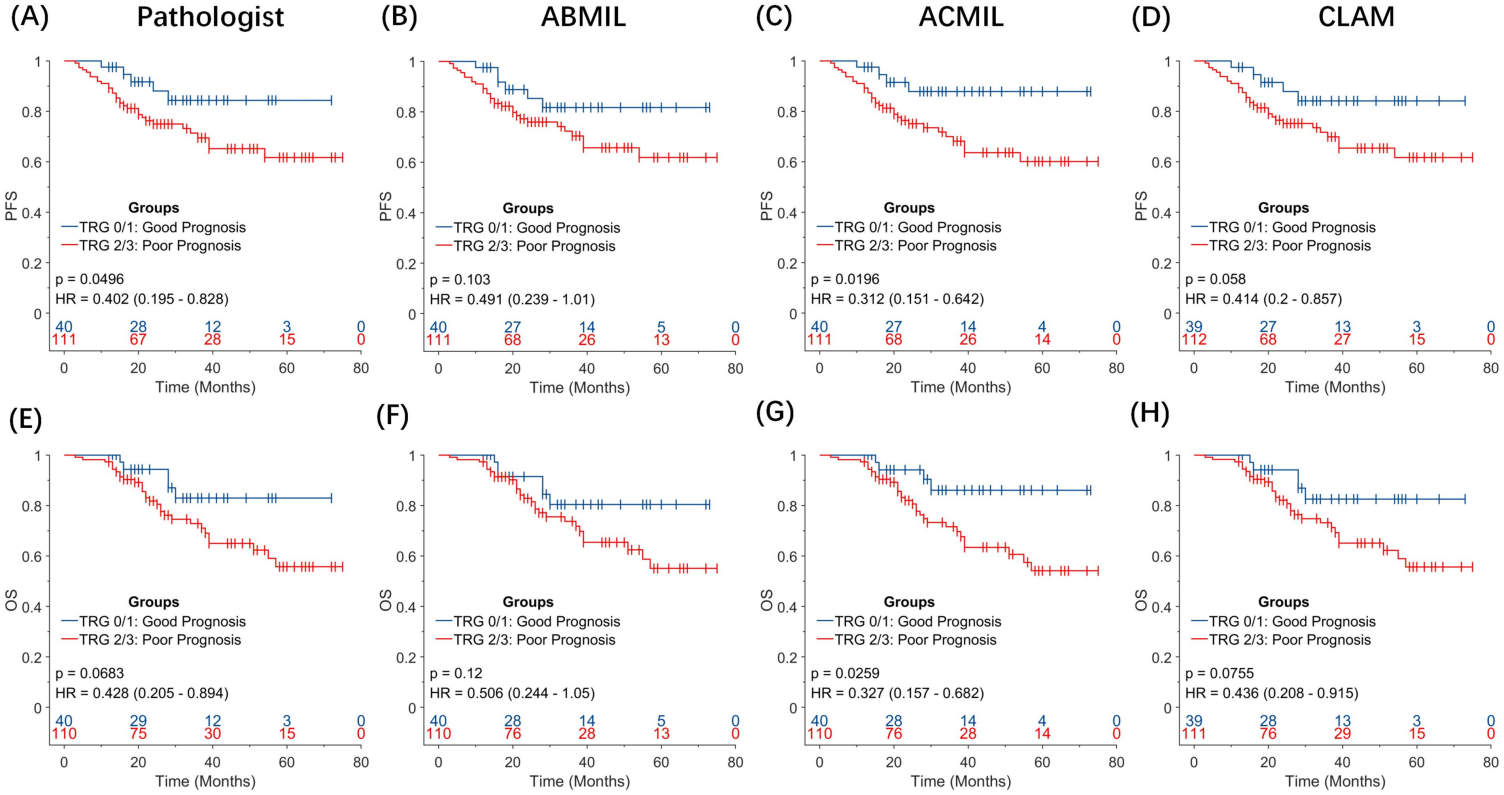
Prognostic significance of pathologist-assigned and artificial intelligence-predicted tumor grade. Kaplan–Meier survival curves for overall survival (OS) and progression-free survival (PFS) stratified by TRG 0–1 (favorable response) versus TRG 2–3 (poor response). **(A,E)** Pathologist-assigned TRG score for PFS and OS. **(B,F)** TRG predictions using the ABMIL model. **(C,G)** TRG predictions using the ACMIL model. **(D,H)** TRG predictions using the CLAM model. Among all comparisons, the ACMIL model **(C,G)** provides the most statistically significant prognostic stratification (PFS: *p* = 0.0196; OS: *p* = 0.0259), surpassing both manual pathologist grading (OS: *p* = 0.0683) and other MIL approaches.

To assess the prognostic relevance of TRG in EC, we examined whether pathologist-assigned or AI-predicted TRG score could stratify patients by long-term survival (Figure 5). Given the clinical convention that treatment responders exhibit lower TRG score, the patients were classified into two groups: TRG 0–1 (favorable response) and TRG 2–3 (poor response). Kaplan–Meier survival curves were generated for PFS and OS, and survival differences were evaluated using the log-rank test. Hazard ratios (HRs) and 95% CIs were estimated using Cox proportional hazard regression.

When pathologist-assigned TRG score was used, the difference in OS between the TRG 0–1 and TRG 2–3 groups was not statistically significant (log-rank *p* = 0.068; HR = 0.428, 95% CI: 0.205–0.894).

In contrast, the AI-predicted TRG score, particularly those generated using the ACMIL model, demonstrated an improved ability to stratify patient outcomes. For PFS, the predicted TRG 0/1 group showed a sustained advantage over the predicted TRG 2/3 group throughout the follow-up period [HR = 0.312, 95% confidence interval (CI), 0.151–0.642; *p* = 0.0196]. A consistent pattern was observed in OS. Patients with predicted TRG 0/1 achieved longer OS than those with predicted TRG 2/3 (HR = 0.327, 95% CI: 0.157–0.682, *p* = 0.0259).

These results suggest that AI-derived TRG score captures the histopathological correlates of treatment response more effectively than conventional grading, providing superior discrimination of long-term outcomes. Specifically, ACMIL-predicted TRG has emerged as a robust surrogate biomarker for patient prognosis after NAT, offering potential value for response assessment and clinical trial endpoint refinement.

## 4 Discussion

In this study, we developed and validated a weakly supervised AI framework for the automated assessment of TRG in EC following NAT. Through combining large-scale pathology foundation models with MIL, the proposed approach achieved accurate, efficient, and reproducible patient-level TRG score prediction without the need for manual region annotations. The results demonstrated high consistency between AI-predicted and pathologist-assigned TRG score. Notably, the AI-derived TRG score exhibited superior prognostic value for PFS and OS. These findings highlight the potential of weakly supervised pathological AI systems in enhancing the objectivity and clinical utility of histopathological response assessments.

Conventional TRG evaluation remains the standard method for assessing histopathological responses after NAT; however, it is inherently limited by subjectivity, inter-observer variability, and workload intensity. The emergence of digital pathology and AI has provided new opportunities to overcome these challenges. Previous studies, such as those by Tolkach et al. (17) and Wang et al. (18), have demonstrated the feasibility of AI-assisted TRG evaluation in esophageal tumors. However, these approaches rely on manual patch-level labeling or continuous tumor percentage estimation, restricting scalability and clinical adoption.

Our framework overcomes these limitations through a fully weakly supervised design in which each patient is modeled as a “bag” containing multiple WSIs as instances. The model was trained directly on patient-level TRG labels that are readily available in clinical pathology reports, eliminating the need for manual slide- or pixel-level annotations. This strategy significantly reduces data preparation costs and enhances the feasibility of applying AI models to large-scale real-world cohorts.

The adoption of pathology foundation models played a central role in our framework. By evaluating three recent slide-level pretrained models, we identified CHIEF as the most discriminative and robust backbone for feature extraction at the slide level. The hierarchical vision transformer structure of CHIEF enables the simultaneous encoding of fine-grained cellular features and global tissue architecture, which are crucial for accurately characterizing tumor regression after NAT. These findings emphasize that model selection in computational pathology should be task-specific. Although Prov-Gigapath offers scalability through patch-level robustness, it lacks contextual integration. In contrast, CHIEF captures tissue-level organization more effectively, which is critical for TRG differentiation. This systematic comparison also contributes to a broader understanding of how foundation models can be leveraged and optimized for downstream clinical applications.

Building on CHIEF embeddings, we implemented and compared three representative MIL algorithms to integrate multiple WSIs per patient into a unified predictive model. Although the field is rapidly evolving with newer histology-focused MIL approaches, such as the method described in reference (27), which further optimize feature aggregation, we focused on established methods, including CLAM and ACMIL, to ensure robust reproducibility. In our comparative analysis, ACMIL achieved the best overall balance between accuracy and stability, reaching an accuracy of 82.7% and a macro-average AUC of 0.81 under five-fold cross-validation. Furthermore, confusion matrix analysis revealed that most classification errors occurred between adjacent TRG categories, reflecting the biological continuity of tumor regression rather than random misclassification.

Notably, AI-derived TRG score demonstrated stronger prognostic power than manual grading did. AI-predicted TRG (0–1 vs. 2–3) was used to effectively stratify patients according to PFS and OS, whereas the pathologist-assigned grades were not statistically significant. This superior prognostic stratification suggests that the AI model captures underlying histological complexities that extend beyond human visual perception, a conclusion validated within the emerging quantitative pathology paradigm. Li et al. (28) demonstrated that quantitative descriptors of tissue complexity, such as structural entropy, can provide biologically meaningful information about tumor structure by establishing an entropy-based comprehensive measurement framework in digital pathology. Li (29) improved interpretability in weakly supervised settings by decoding the potential spatial relationships between cells from pathological images through extracting spatial descriptors. Although manual TRG grading mainly quantifies the percentage of residual tumor burden, spatial tissue organization and tissue complexity are also key determinants of prognosis. The AI model can sensitively capture this information for prognostic prediction. Additionally, the MIL framework proposed in this study integrates the global information from multiple whole-slide images of the same patient, effectively capturing the spatial heterogeneity of individual patients. This heterogeneity, although known to have a significant impact on prognosis, is underestimated in manual TRG score. Clinically, AI-assisted TRG score could achieve standardized, objective and reproducible response assessments. Moreover, AI can capture the continuous biological spectrum of pathological responses. The TRG score predicted by AI are not a strictly ordered classification but a continuous severity grade, which can provide a more refined and biologically accurate reflection of treatment effects. This advantage helps to reduce inter-observer variability and facilitate multicenter clinical trials. Furthermore, the AI framework can provide more accurate survival stratification, offering information for individualized postoperative management decisions, including the need for adjuvant therapy or intensified surveillance in high-risk patients.

This study has several methodological and translational strengths. First, it introduces a fully weakly supervised paradigm for TRG assessment using only patient-level labels, eliminating the dependence on detailed annotations. Second, it leverages the representational capacity of pathology foundation models, allowing generalization to diverse histopathological patterns. Third, the model was prognostically validated, establishing its potential as a diagnostic tool and biomarker of clinical outcomes. Collectively, these advances provide a scalable and explainable framework suitable for real-world clinical deployment.

Despite its promising performance, this study has some limitations that highlight the directions for future studies. The model was developed and validated using data from a single medical center, which may limit its external generalizability owing to variations in slide preparation, staining, and scanning protocols across institutions. Although the foundation model (CHIEF) utilized in this study—pretrained on over 1 million diverse slides—provides inherent feature robustness against such variations, domain shifts caused by different scanners and staining intensities remain a potential challenge. Future studies should not only involve large-scale, multicenter validations but also incorporate rigorous stain normalization and domain adaptation techniques to ensure consistent performance across diverse clinical settings. The lack of multi-factor adjustment is another limitation. With larger sample sizes, subsequent research should assess the independent AI-TRG prognostic value while adjusting for key covariates such as ypT, ypN, and staging. Finally, the relatively small cohort of 157 patients, although cross-validated, underscores the need for larger datasets to further improve stability and representational capacity.

We proposed a weakly supervised AI framework for the automated assessment of TRG in post-NAT for EC. This framework performs well in classification accuracy and exhibits superior prognostic stratification compared with conventional manual assessment. By integrating the pathology foundation model with advanced MIL paradigms, our study provides a practical solution to the current challenges of labor-intensive annotations and subjective variability in TRG assessments. In addition to its direct clinical applicability for the precise diagnosis and treatment of EC, our study offers a methodological reference for similar pathological image analysis tasks in other cancer types that rely on patient-level weak labels.

## Data Availability

The original contributions presented in the study are included in the article/supplementary material, further inquiries can be directed to the corresponding authors.
